# Feasibility and exploratory assessment of large language models for pediatric dentistry queries: a comparative study

**DOI:** 10.3389/froh.2026.1813936

**Published:** 2026-04-24

**Authors:** Sanjeev B. Khanagar, Ali Al-Ehaideb, Nouf Almutairi, Layan Alqahtani, Prabhadevi C. Maganur, Satish Vishwanathaiah, Audrey Madonna Dcruz, Kiran Iyer

**Affiliations:** 1 Preventive Dental Science Department, College of Dentistry, King Saud bin Abdulaziz University for Health Sciences, Riyadh, Saudi Arabia; 2 King Abdullah International Medical Research Centre, Ministry of National Guard Health Affairs, Riyadh, Saudi Arabia; 3 Ministry of the National Guard Health Affairs, Riyadh, Saudi Arabia; 4 College of Dentistry, King Saud bin Abdulaziz University for Health Sciences, Riyadh, Saudi Arabia; 5 Division of Pediatric Dentistry, Department of Preventive Dental Sciences, College of Dentistry, Jazan University, Jazan, Saudi Arabia; 6 Department of Public Health Dentistry, AB Shetty Memorial Institute of Dental Sciences, Nitte (Deemed to be University), Karnataka, India

**Keywords:** accuracy, artificial intelligence, assessment, ChatGPT, health information, large language models, pediatric dentistry, quality

## Abstract

**Background:**

Large Language Models (LLMs) are increasingly used by caregivers to obtain pediatric health information. However, concerns persist regarding the accuracy, reliability, and readability of AI-generated content, especially in pediatric dentistry, where caregiver comprehension is crucial.

**Objective:**

To conduct an exploratory feasibility assessment of evaluating accuracy, quality, reliability, and readability of responses generated by ChatGPT-4, Google Gemini, and DeepSeek to common pediatric dentistry queries.

**Methods:**

This exploratory comparative cross-sectional feasibility study utilized 15 patient-oriented pediatric dentistry questions identified through structured searches and expert screening. Each question was submitted verbatim to ChatGPT-4, Gemini, and DeepSeek under standardized conditions. Responses were independently evaluated by three calibrated pediatric dentistry experts using the Global Quality Scale (GQS), a modified DISCERN tool, and the Accuracy of Information Index (AOI). Readability was assessed using the Flesch Reading Ease Score (FRES) and the Flesch–Kincaid Grade Level (FKGL). Inter-examiner reliability was assessed using intraclass correlation coefficients (ICC). Statistical comparisons between LLMs were performed using a fixed-effects model with *post-hoc* pairwise analysis. Inter-examiner agreement was further evaluated using Bland–Altman analysis. A *p*-value of <0.05 was considered statistically significant.

**Results:**

Overall scoring was consistent across examiners, with minor variability observed across domains. A linear mixed-effects model conducted separately for each domain demonstrated that LLM type significantly influenced GQS scores (F = 7.90, *p* = 0.00), with Gemini and DeepSeek outperforming ChatGPT. No significant differences were observed for AOI (*p* = 0.44) and DISCERN (*p* = 0.06). Bland-Altman analysis indicated minimal inter-examiner bias; however, the limits of agreement were relatively wide considering the scale range, reflecting variability between individual ratings. Single-measure ICC demonstrated poor agreement (ICC = 0.26), while higher reliability observed when scores were averaged (ICC = 0.90).

**Conclusion:**

This study offers an exploratory feasibility assessment of LLM evaluation in pediatric dentistry. While the models generally produced high-quality outputs, variations in accuracy, readability, and significant inter-examiner variability highlight important methodological challenges. These findings represent preliminary groundwork and require validation in larger, clinically diverse, real-world settings. LLMs may serve as supportive informational tools; however, their outputs should be interpreted cautiously and used to complement, not replace professional clinical judgment.

## Introduction

Pediatric dentistry is a specialized branch of dentistry focused on the prevention, diagnosis, and management of oral health conditions in children from infancy through adolescence, including those with special health care needs (1, 2). With the growing availability of digital health resources, parents and caregivers often turn to online platforms to obtain dental information that guides decision-making about their children's oral health. Search engines, social media platforms, and video-sharing websites have become primary sources of this information, despite variability in quality and reliability (3, 4).

Recent advances in artificial intelligence (AI), particularly in generative AI and large language models (LLMs), have revolutionized the way health information is accessed and delivered. LLMs such as ChatGPT-4, Google Gemini, and DeepSeek generate human-like responses to user queries by leveraging extensive training on diverse textual datasets (5–9). Unlike traditional search engines, these tools provide synthesized answers in real time, offering a convenient alternative for individuals seeking rapid health-related guidance.

The increasing reliance on LLMs for medical and dental information raises significant concerns about the accuracy, reliability, and appropriateness of the content generated. Previous studies have shown that AI-generated responses may contain factual inaccuracies, incomplete information, or misleading recommendations, which could pose potential risks to patient safety—especially in pediatric healthcare, where caregivers often lack professional medical knowledge (10–14). Therefore, several authors have emphasized that LLMs should be used cautiously as adjunctive tools rather than definitive sources of clinical information (15, 16).

Despite the growing use of LLMs by patients and caregivers, there is a lack of evidence evaluating the quality and trustworthiness of AI-generated responses specifically within the field of pediatric dentistry. Assessing the accuracy, reliability, and readability of such content is crucial, as these factors directly impact caregiver understanding, decision-making, and adherence to professional dental advice (17, 18).

Given the absence of standardized evaluation protocols for conversational AI in pediatric dentistry, this study serves as a feasibility assessment to identify methodological challenges—particularly those related to reproducibility, inter-examiner reliability, and outcome measurement—that must be addressed before definitive comparative effectiveness studies can be conducted. Therefore, this study aimed to conduct an exploratory feasibility assessment of evaluating the accuracy, quality, reliability, and readability of responses generated by ChatGPT-4, Google Gemini, and DeepSeek to common pediatric dentistry queries.

## Materials and methods

### Study design and ethical considerations

A comparative cross-sectional study design was employed. Institutional approval to conduct the study was obtained from the Research Office of the King Abdullah International Medical Research Center (KAIMRC), Riyadh, Saudi Arabia (IRB Approval No. 0000038125; Study No. NRR25/117/2; approved on 1 March 2025). A formal ethical review was waived because the study did not involve human participants, patient data, or animal subjects. This study was conducted in accordance with the CHART (Chatbot Assessment Reporting Tool) 2025 guidelines for reporting AI chatbot evaluations in healthcare. A completed CHART checklist is provided as Supplementary Material (Supplementary Table 1).

**Figure 1:**
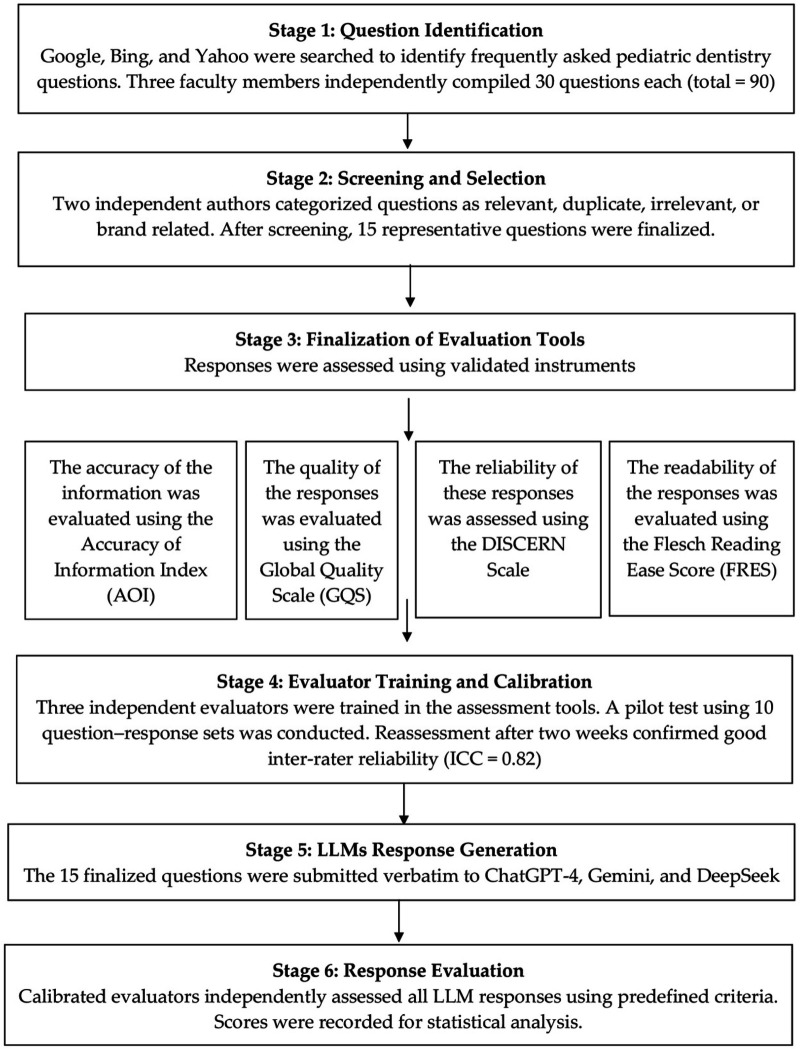
Flow chart for methodology.

The study methodology consisted of six sequential stages: Flowchart for Methodology (Figure 1)

Stage 1: Identification of Patient-Oriented Questions: Three academic faculty members (S.B.K., K.I., and A.A.E), each with substantial research experience, independently conducted online searches using Google, Bing, and Yahoo. Websites offering patient-oriented dental information were screened to identify frequently asked questions related to pediatric dentistry. Each investigator compiled a list of 30 commonly encountered questions, resulting in an initial pool of 90 questions.
Stage 2: Compilation and Screening of Questions: Two independent authors (N.A. and L.A.), who were not involved in collecting the questions, reviewed and categorized them as relevant, duplicate, irrelevant, or brand related. Questions deemed irrelevant to pediatric dentistry, duplicates, or promotional were excluded. After this screening process, 15 representative questions were finalized for analysis. The selection of 15 questions was conducted through a structured, multi-stage process involving independent searches, screening, and expert validation to ensure that the final set was representative of commonly encountered, clinically relevant, patient-oriented pediatric dentistry queries. The goal was to prioritize depth, relevance, and standardized evaluation over quantity, enabling consistent comparisons across models using validated assessment tools (Table 1).
Stage 3: Finalization of Evaluation Instruments:
Accuracy Assessment: Response accuracy was evaluated using the Accuracy of Information Index (AOI) (19), which comprises six criteria: factual accuracy, corroboration with established knowledge, internal consistency, clarity, specificity, and relevance. Each criterion was scored on a 3-point scale (0 = poor, 1 = moderate, 2 = excellent), resulting in a maximum possible score of 12 per response.
Quality Assessment: The Global Quality Scale (GQS) was used to assess the overall quality and usefulness of responses for patients (20, 21). Scores ranged from 1 (poor quality, limited usefulness) to 5 (excellent quality and coherence). Scores of 1–2 were categorized as low quality, 3 as moderate quality, and 4–5 as high quality.
Reliability Assessment: Response reliability was evaluated using a modified 8-item DISCERN instrument, adapted from the original DISCERN tool and previously applied in digital health information studies (21, 22). The modified scale assessed clarity of objectives, relevance, sources of information, timeliness, balance and impartiality, acknowledgment of uncertainty, achievement of objectives, and availability of additional support resources. Each item was scored on a scale from 1 (no information) to 5 (complete information), yielding a total score range of 8 to 40. Scores were categorized as poor (8–15), moderate (16–31), or good (32–40).
Readability Assessment: Readability was assessed using the Flesch Reading Ease Score (FRES) (23, 24). Readability of the response texts was assessed using the Flesch Reading Ease Score (FRES) calculated with an online readability calculator (https://readability.ch/en/). The Flesch formula, developed by Rudolf Flesch (1948), was applied as: FRE = 206.835−(1.015 × ASL)−(84.6 × ASW), where ASL represents average sentence length and ASW represents average syllables per word. Scores range from 0 to 100, corresponding to very hard to extremely easy readability levels.
Stage 4: Evaluator Training and Calibration: Three independent evaluators (A.A.E, P.C.M., and S.V.), each holding a master's degree in pediatric dentistry and possessing academic research experience, were trained in the use of all evaluation tools. The evaluators were blinded to the identity of the LLM generating each response. A pilot calibration exercise was conducted using 10 randomly selected question–response sets. Reassessment after a two-week interval demonstrated good inter-rater reliability, with an intraclass correlation coefficient (ICC) of 0.82. Any discrepancies were resolved through consultation with a fourth evaluator (A.M.D.).
Stage 5: LLM Query and Response Generation: A single author (N.A.) submitted the finalized questions verbatim to the publicly available versions of ChatGPT-4 (OpenAI), Google Gemini (Google DeepMind; web interface, version available as of 15 October 2025), and DeepSeek (DeepSeek AI; web interface). All queries were performed on the same day using a single device to minimize temporal and system-related variability (15 October 2025, Riyadh, Saudi Arabia). All questions were submitted using a one-shot prompting approach without follow-up interactions. Queries were performed using the default web interface settings of each LLM, without enabling additional tools such as browsing or external integrations, to ensure consistency and comparability across models. The responses generated were documented exactly as received and organized for future evaluation. The model specification was refined iteratively to appropriately account for the nested structure of the data. This analytical flexibility reflects the exploratory nature of the study and the lack of established statistical frameworks for evaluating LLMs in this context.
Stage 6: Response Evaluation and Data Entry: Three calibrated evaluators (A.A.E, P.C.M., and S.V.) independently assessed the responses generated by the LLMs using predefined evaluation instruments. All recorded scores and observations were systematically entered into a Microsoft Excel spreadsheet for data management and subsequent statistical analysis.

**Table 1:** List of prompts submitted to the LLMs.

S. No.	Prompt Submitted to LLMs
1	Why are primary (baby) teeth important for my child?
2	When should my child have their first dental visit?
3	How should I clean my baby's teeth and gums?
4	Are thumb sucking and pacifier habits harmful to my child's teeth?
5	What foods increase the risk of cavities in children?
6	When should I start brushing my child's teeth with toothpaste?
7	Until what age should I help my child brush their teeth?
8	When do baby teeth normally fall out?
9	How is a pediatric dentist different from a general dentist?
10	Does my child need dental sealants?
11	What should I do if my child has a toothache?
12	How can I prevent cavities in my child?
13	Is fluoride safe and necessary for children?
14	How often should my child visit the dentist?
15	What are early signs of tooth decay in children?

### Statistical analysis

The examiners recorded their data using Microsoft Excel (Microsoft Corp., New York, NY, USA), which was subsequently analyzed with the Statistical Package for the Social Sciences (SPSS) software, version 20 (2011; IBM Corp., Armonk, NY, USA). Descriptive statistics are presented as mean ± standard deviation (Mean ± SD) scores assigned by examiners for each LLM based on GQS, DISCERN, and AOI. Linear mixed-effects models were conducted separately for each outcome domain, with LLM as a fixed effect, question-level units included as random intercepts, and examiners treated as repeated measures. Inter-examiner agreement was evaluated using Bland-Altman analysis and intraclass correlation coefficients (ICC). Bonferroni-adjusted pairwise comparisons were also performed. Additionally, inter-examiner agreement and bias were further assessed using Bland-Altman analysis. Differences in Flesch Reading Ease and Flesch-Kincaid grade levels between LLMs were analyzed using ANOVA followed by Bonferroni *post-hoc* tests. A *p*-value of <0.05 was considered statistically significant for all analyses.

## Results

Intra-examiner scoring was consistent and similar across all LLMs. Examiner 1's Global Quality Scores and DISCERN scores were slightly higher across the LLMs. Variation in intra-examiner scoring was primarily observed in the Accuracy of Information Index (AOI), particularly between examiners 1 and 2, with Gemini outperforming the other two LLMs. Regarding inter-examiner consistency, examiner 3 demonstrated greater scoring consistency, as indicated by a lower standard deviation, compared to the other examiners across all three LLMs (Table 2).

**Table 2:** Mean scores based on examiners’ assessments of the fifteen questions across the three LLMs.

GQS	DISCERN	AOI	GQS	DISCERN	AOI	GQS	DISCERN	AOI
ChatGPT Mean (±S.D) Examiner 1			ChatGPT Mean (±S.D) Examiner 2			ChatGPT Mean (±S.D) Examiner 3		
4.47 (0.64)	4.33 (0.61)	9.47 (0.91)	4.47 (0.51)	4.40 (0.91)	8.73 (1.38)	4.20 (0.56)	4.07 (0.45)	9.07 (0.59)
Gemini Mean (± S.D) Examiner 1			Gemini Mean (± S.D) Examiner 2			Gemini Mean (± S.D) Examiner 3		
4.67 (0.51)	4.60 (0.50)	9.20 (0.77)	4.80 (0.41)	4.67 (0.61)	9.40 (1.12)	4.87 (0.35)	4.47 (0.64)	9.47 (0.83)
DeepSeek Mean (S.D) Examiner 1			DeepSeek Mean (S.D) Examiner 2			DeepSeek Mean (S.D) Examiner 3		
4.60 (0.63)	4.40 (0.63)	8.73 (1.03)	4.87 (0.35)	4.07 (0.45)	8.93 (0.70)	4.80 (0.41)	4.27 (0.59)	9.47 (0.64)

The linear mixed-effects models were conducted separately for each outcome domain (AOI, DISCERN, and GQS), with LLM as a fixed effect, question-level units specified as random intercepts, and examiners treated as repeated measures (Table 3).

**Table 3:** Fixed effect analysis with *post-hoc* pairwise comparison of LLMs.

Fixed effects and estimated means								
DOMAIN	LLM	Mean ± S E	95% CI		F	df	P Value	n^2^ (Effect Size)
			Lower	Upper				
GQS	ChatGPT	4.38 ± 0.09	4.20	4.55	7.89	40	0.00*	0.28
	DeepSeek	4.79 ± 0.09	4.62	4.96				
	Gemini	4.80 ± 0.09	4.62	4.97				
AOI	ChatGPT	9.09 ± 0.17	8.75	9.43	0.83	37	0.44	0.04
	DeepSeek	9.16 ± 0.17	8.82	9.50				
	Gemini	9.39 ± 0.17	9.04	9.73				
DISCERN	ChatGPT	4.25 ± 0.10	4.04	4.46	3.02	40	0.06	0.13
	DeepSeek	4.26 ± 0.10	4.05	4.47				
	Gemini	4.57 ± 0.10	4.36	4.78				
**Pairwise comparison between LLMs (Bonferroni Adjusted)**								
DOMAIN	LLM Comparison	Mean Difference	95% CI		*P* Value			
			Lower	Upper				
**GQS**	ChatGPT vs DeepSeek	−0.42	−0.72	−0.12	0.00*			
	ChatGPT vs Gemini	−0.42	−072	−0.12	0.00*			
	DeepSeek vs Gemini	−0.01	−0.31	0.30	1.00			
**AOI**	ChatGPT vs DeepSeek	−0.07	−0.67	0.53	1.00			
	ChatGPT vs Gemini	−0.30	−0.89	0.30	0.67			
	DeepSeek vs Gemini	−0.22	−0.82	0.38	1.00			
**DISCERN**	ChatGPT vs DeepSeek	−0.01	−0.38	0.36	1.00			
	ChatGPT vs Gemini	−0.32	−0.68	0.05	0.10			
	DeepSeek vs Gemini	−0.31	−0.67	0.06	0.12			

*p < 0.05-significant, S.E – standard error, and CI- confidence interval.

For the GQS domain, a significant effect of the LLM was observed (F = 7.90, *p* = 0.00). Estimated marginal means indicated that DeepSeek (mean = 4.79 ± 0.09) and Gemini (mean = 4.80 ± 0.09) performed similarly. *post-hoc* pairwise comparisons with Bonferroni adjustment demonstrated that ChatGPT scores were significantly lower than those of both DeepSeek (mean difference = −0.42, *p* = 0.00) and Gemini (mean difference = −0.42, *p* = 0.01). No significant difference was observed between DeepSeek and Gemini (*p* = 1.00) (Table 3).

For the AOI domain, no statistically significant differences were observed between LLMs (F = 0.83, *p* = 0.44), despite the Gemini LLM demonstrating the highest mean score (Table 3).

Similarly, for the DISCERN domain, no statistically significant differences were identified between the LLMs (F = 3.02, *p* = 0.06), although the Gemini LLM showed slightly higher mean scores compared to the other models (Table 3).

Bland-Altman analysis was performed to assess inter-examiner agreement. The mean differences (bias) between examiners were close to zero across all comparisons, indicating minimal systematic differences. Examiner 1 and Examiner 2 demonstrated a mean difference of 0.02, with lower and upper limits of agreement at −1.59 and 1.62, respectively. Similarly, Examiner 1 and Examiner 3 showed a mean difference of −0.02, with lower and upper limits of agreement at −1.82 and 1.78, respectively. Finally, the agreement between Examiner 2 and Examiner 3 exhibited a mean difference of −0.04, with lower and upper limits of agreement at −1.64 and 1.56, respectively.

When interpreted relative to the GQS scale range, these limits of agreement (±1.6 to ±1.8) represent approximately 40% to 45% of the total scale, indicating considerable variability between individual examiner ratings. Similarly, wide limits were observed across the other outcome domains when interpreted against their respective scale ranges.

**Figure 2:**
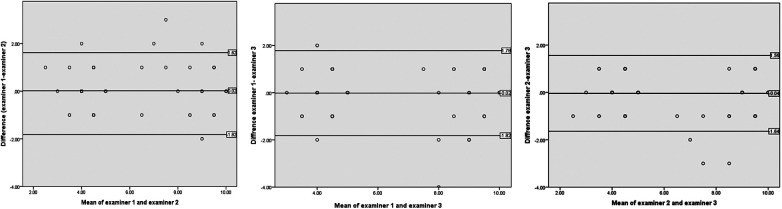
Bland-Altman plot illustrating the agreement and bias between examiners. - **Plot A** (*X*-axis): Mean of Examiner 1 and Examiner 2 - **Plot B** (*X*-axis): Mean of Examiner 1 and Examiner 3 - **Plot C** (*X*-axis): Mean of Examiner 2 and Examiner 3. - **Plot A** (*Y*-axis): Difference between Examiner 1 and Examiner 2 - **Plot B** (*Y*-axis): Difference between Examiner 1 and Examiner 3 - **Plot C** (*Y*-axis): Difference between Examiner 2 and Examiner 3. - Central Line – Mean - Central Line – Mean, Above: Upper Limit, Below: Lower Limit.

These findings are consistent with a low single-measure intraclass correlation coefficient (ICC = 0.26), indicating poor reliability at the individual examiner level. In contrast, reliability improved when scores were averaged (ICC = 0.90) (Table 4) and (Figure 2).

**Table 4:** Bland-Altman analysis to evaluate inter-examiner agreement and bias.

Comparison between Examiners	Mean Difference (Bias)	Standard Deviation	Lower Limit of Agreement	Upper Limit of Agreement
Examiner 1 vs Examiner 2	0.02	0.82	−1.59	1.62
Examiner 1 vs Examiner 3	−0.02	0.92	−1.82	1.78
Examiner 2 vs Examiner 3	−0.04	0.81	−1.64	1.56

Limits of agreement were calculated as mean difference ±1.96 × standard deviation.

The reliability of examiners’ grading was analyzed using the Intraclass Correlation Coefficient (ICC). The results revealed poor reliability for individual examiners based on single measures (ICC=0.26, 95% CI: 0.14–0.48). However, the average measures (mean of examiner scores), justified by a significant *F*-test (*p* < 0.001), demonstrated excellent reliability in grading between examiners (ICC = 0.90, 95% CI: 0.82–0.96). While average-measure ICC indicated excellent reliability, the low single-measure ICC reflects variability between individual examiners, suggesting that aggregated scoring provides more stable estimates (Table 5).

**Table 5:** Evaluation of inter-rater reliability using intra-class correlation (ICC) statistics.

Type of Measure	Intra Class Correlation	95% Confidence Interval		F Test Value	Sig
		Lower Bound	Upper Bound		
Single Measures	0.26	0.14	0.48	11.02	0.00*
Average Measures	0.90	0.82	0.96		

* Significance- *P* < 0.05.

Analysis of variance (ANOVA) and Bonferroni *post hoc* tests were conducted to assess significant differences in readability ease and readability grade level among the three LLMs, based on the Flesch scoring system. Readability grade level varied significantly (F = 10.13, *p* < 0.001) between the LLMs, whereas no significant difference was found in readability ease among the three LLMs (Table 6).

**Table 6:** Analysis of significance for flesch Reading ease and flesch-kincaid grade level between LLMs using ANOVA and Bonferroni *post-hoc* tests.

Between LLM Groups	Sum of Squares	Mean Square	F	df	Sig.	
Flesch Reading Ease	499.36		249.68	2.84	2	0.70
Flesch Kincad Grade Level	46.38		23.19	10.13	2	0.00*
*Significance- *P* < 0.05						
Bonferroni *post-hoc* Analysis						
Dependent Variable	LLM Group	LLM Comparison Group	Mean Difference	Sig.	95% Confidence Interval	
					Lower Bound	Upper Bound
Flesch Reading Ease	ChatGPT	Gemini	7.51	0.10	−1.02	16.04
		DeepSeek	1.00	1.00	−7.53	9.53
	Gemini	ChatGPT	−7.51	0.10	−16.04	1.02
		DeepSeek	−6.51	0.19	−15.04	2.02
	DeepSeek	ChatGPT	−1.00	1.00	−9.53	7.53
		Gemini	6.51	0.19	−2.02	15.04
Flesch Kincad Grade Level	ChatGPT	Gemini	−2.24	0.00*	−3.62	−0.86
		DeepSeek	−0.20	1.00	−1.57	1.17
	Gemini	ChatGPT	2.24	0.00*	0.86	3.62
		DeepSeek	2.04	0.00*	0.66	3.42
	DeepSeek	ChatGPT	0.20	1.00	−1.17	1.57
		Gemini	−2.04	0.00*	−3.42	−0.66

*The mean difference is significant at the 0.05 level.

.

Bonferroni *post hoc* analysis was conducted to further examine which LLM differed based on grade level. Gemini's readability grade was consistently and significantly higher than that of ChatGPT-4 (*p* = 0.00) and DeepSeek (*p* = 0.00), whereas ChatGPT-4 and DeepSeek had nearly identical readability grade levels. This indicates that the responses generated by ChatGPT-4 and DeepSeek are easier for end readers to comprehend compared to Gemini, which uses more sophisticated terminology and sentence structures (Table 6).

## Discussion

These preliminary findings suggest that contemporary LLMs demonstrate comparable performance characteristics on standard FAQ queries. However, significant methodological considerations related to response variability and assessment reliability have been identified, warranting attention in future confirmatory studies.

The present study aimed to conduct an exploratory feasibility assessment of evaluating the accuracy, quality, reliability, and readability of responses generated by ChatGPT-4, Google Gemini, and DeepSeek to common pediatric dentistry queries. Overall, all three LLMs demonstrated high Global Quality Scale (GQS) and DISCERN scores, indicating that the responses were generally useful and scientifically sound. However, variability was observed in accuracy scores (AOI), particularly with ChatGPT-4, and significant differences were found in readability grade levels among the models. While quality and reliability indicators did not differ significantly across LLMs, readability grade levels varied notably, with Gemini producing responses at a higher educational level compared to ChatGPT-4 and DeepSeek.

In the present study, no statistically significant differences were observed in GQS and DISCERN scores among the three LLMs, indicating that all models produced responses of comparable overall quality and reliability. The consistently high GQS scores (mean > 4) suggest that the responses were well-structured, clinically relevant, patient-oriented, and generally comprehensive. Similarly, DISCERN scores reflected acceptable scientific accuracy, clarity of objectives, and a balanced presentation of information. These findings indicate that contemporary LLMs can synthesize pediatric dental information at a level comparable to that of professional patient education materials.

However, findings from previous studies reveal considerable variability in the performance of large language models (LLMs) within pediatric dentistry. Mukhopadhyay A et al. (25) evaluated ChatGPT-4, Claude 3.5 Sonnet, and DeepSeek R1 using multiple-choice questions (MCQs) and identified statistically significant differences in both accuracy and the quality of justifications. DeepSeek R1 achieved the highest overall performance, demonstrating superior comprehension and contextual reasoning. The authors suggested that newer-generation models may be particularly well suited for specialized domains such as dentistry, highlighting the rapid evolution of LLM capabilities.

In contrast, other studies have highlighted the strengths of ChatGPT in specific clinical contexts. Buldur M et al. (26) demonstrated that ChatGPT provides reliable information regarding fluoride use, emphasizing its potential value in patient education. Similarly, Maltarollo TFH et al. 27 recognized ChatGPT as a useful adjunct for pediatric dentists but stressed the importance of cautious and critical application. Dermata A et al. (28) further reported that ChatGPT-4.0 was the most trustworthy LLM within their evaluation framework, while clearly emphasizing that artificial intelligence should complement rather than replace professional clinical expertise.

In the present study, Flesch Reading Ease scores did not differ significantly among LLMs; however, the LLMs, Flesch–Kincaid Grade Level showed a significant difference (F = 10.13, *p* < 0.001). Gemini generated responses at a higher reading grade level compared to ChatGPT and indicating the use of more complex language and structures. In pediatric dentistry, this distinction is significant because caregivers may possess varying levels of health literacy. Materials written above recommended readability thresholds can reduce comprehension and adherence to preventive advice (29). As patient education resources, ChatGPT and DeepSeek may be more suitable for communicating with caregivers, whereas responses from Gemini may require further simplification. A study conducted by Raj M et al. (30) compared the clinical quality, readability, and originality of LLM-generated responses to pediatric dental inquiries. Their findings revealed that ChatGPT-4o demonstrated superior overall performance, whereas Gemini 2.0 exhibited the greatest variability in readability grade levels, likely due to inconsistencies in sentence structure and lexical choice. In contrast, ChatGPT models showed stable linguistic performance, with readability levels consistently within the collegiate range, making them particularly suitable for educated caregivers and early-career dental professionals seeking clinical guidance (31).

Notably, these findings closely align with the results of our study. While quality and reliability indicators did not differ significantly among the evaluated LLMs, marked differences were observed in readability grade levels. Specifically, Gemini generated responses at a higher educational level compared to ChatGPT-4 and DeepSeek. Collectively, the existing evidence suggests that although multiple LLMs demonstrate promising clinical potential, variations in model architecture and linguistic output can substantially influence readability and practical applicability. This underscores the importance of context-specific evaluation and careful model selection when integrating LLMs into pediatric dental education and patient communication. The present study was limited to text-based queries and did not evaluate multimodal capabilities of LLMs. Recent studies have demonstrated that multimodal LLMs show variable performance in dental image interpretation tasks, including anatomical landmark identification and diagnostic reasoning (32). Future research should incorporate image-based and multimodal evaluations—such as caries detection, dental trauma assessment, and eruption analysis—to better reflect real-world clinical applications and fully assess the capabilities of next-generation AI systems (33). While recent studies have evaluated newer-generation models such as ChatGPT-4o and Gemini 2.0, the present study assessed versions of LLMs that were publicly accessible and stable at the time of data collection. Given the rapid pace of development in AI systems, differences in model architecture and updates may influence performance; therefore, direct comparisons across studies should be interpreted with caution.

Collectively, these findings highlight that while LLMs show considerable promise in pediatric dentistry, their performance varies across different models, applications, and evaluation criteria. This variability underscores the need for context-specific validation and responsible integration into clinical and educational practice. While the present study demonstrates that LLMs can generate high-quality, patient-oriented information, their role in clinical decision-making remains limited. Clinical usefulness in a strict sense requires the ability to perform complex reasoning, contextual interpretation, and evidence-based judgment. Recent studies have begun to evaluate reasoning-capable AI systems in dental and medical contexts, highlighting both their potential and current limitations (34, 35). A key consideration in interpreting the present findings is the potential for training data contamination. The pediatric dentistry questions used in this study were derived from commonly available online sources, which may overlap with the training corpora of the evaluated large language models. Consequently, the observed high accuracy and quality scores may partially reflect retrieval or reproduction of previously learned information rather than purely generative reasoning. While this reflects realistic end-user scenarios, it limits the ability to attribute performance solely to the models’ reasoning capabilities.

The integration of LLMs into clinical contexts requires careful consideration of regulatory, ethical, and patient safety frameworks, which remain areas of ongoing development. The integration of artificial intelligence tools into healthcare settings, especially those accessed directly by patients or caregivers, requires careful consideration of regulatory and medico-legal frameworks. The United States Food and Drug Administration (FDA) classifies certain AI-based health technologies under the Software as a Medical Device (SaMD) framework, which employs a risk-based approach to ensure safety, effectiveness, and clinical validity (36, 37). Although conversational LLMs used for general informational purposes may not currently be subject to strict regulatory classification, their growing use in health-related decision-making raises significant concerns regarding oversight and accountability. In the Saudi Arabian context, the Saudi Food and Drug Authority (SFDA) has initiated the development of regulatory pathways for digital health technologies, emphasizing patient safety, data governance, and clinical reliability (38). However, regulatory frameworks for general-purpose AI tools, such as LLMs remain in an evolving stage, especially when these systems are used outside formal clinical settings.From a medico-legal perspective, the use of AI-generated health information presents challenges related to liability and responsibility. In the absence of clear regulatory oversight, it remains uncertain whether accountability lies with developers, healthcare providers, or end users. This issue is particularly critical in pediatric settings, where caregivers may rely on AI-generated advice for decision-making. Ethical and governance concerns surrounding AI in healthcare have been emphasized by global organizations, underscoring the need for transparency, accountability, and human oversight (39).These considerations underscore the need for cautious interpretation and use of LLM outputs, as well as the development of clearer regulatory guidelines to ensure their safe and effective integration into healthcare practice.

In the present study, various modeling approaches were explored to identify the most appropriate specification for the clustered data structure. The variability in effect size estimates across model specifications highlights the sensitivity of LLM evaluation outcomes to statistical modeling choices. This observed variation underscores the importance of careful statistical design and transparent reporting in AI evaluation studies. The divergence in findings across GQS, AOI, and DISCERN highlights the multidimensional nature of LLM evaluation. While GQS captures overall perceived quality, it is inherently more subjective and may be influenced by examiner interpretation, as reflected in the observed variability and lower single-measure reliability. In contrast, AOI and DISCERN assess more structured aspects of factual accuracy and reliability, which may explain the lack of significant differences across models. These findings suggest that different evaluation instruments capture distinct constructs and should be interpreted collectively rather than in isolation.

The present study demonstrates several methodological strengths, including evaluations performed by multiple blinded examiners, the use of standardized and validated assessment tools (GQS, DISCERN, AOI, and Flesch scores), prompts administered on the same day to minimize time-related variability, and separate query sessions to prevent contextual influence. Furthermore, the structured six-stage design enhances the clarity and reproducibility of the methods employed. However, a few limitations should be acknowledged, firstly only 15 questions were evaluated, which may not fully capture the comprehensive scope of pediatric dentistry. A formal sample size calculation was not performed. The number of questions was determined based on feasibility and consistency with prior LLM evaluation studies; however, this may limit statistical power. The development of the question set presents a significant methodological limitation. Although the questions were selected through a structured, expert-driven process, formal instrument validation methods—such as Delphi consensus procedures, calculation of content validity indices, pilot testing, and caregiver cognitive interviewing—were not conducted. Additionally, domain coverage mapping and item-level statistical analyses (e.g., assessment of floor and ceiling effects) were not performed. Additionally, the limited number and FAQ-style nature of the prompts may introduce ceiling effects when evaluating high-performing large language models. These limitations may impact the representativeness and construct validity of the findings, as the selected questions might not fully encompass the breadth and complexity of pediatric dentistry or effectively differentiate between levels of model performance. Therefore, the results should be interpreted within the context of this limited evaluation framework. Future studies incorporating larger and more diverse question sets, including scenario-based and clinically complex queries, are recommended to enhance discriminatory power.

The second limitation is that this study may be affected by potential training data contamination, as the queries were derived from widely available pediatric dentistry FAQs that could have been included in the models’ training datasets. This may result in an overestimation of performance due to memorization effects. Future research should incorporate novel, expert-generated, or adversarial queries to more accurately assess true reasoning and generalization capabilities.The third limitation is that responses were evaluated at a single time point; however, LLM outputs may vary over times. As such, the findings represent a snapshot evaluation and may not fully reflect longitudinal consistency. The use of a one-shot prompting strategy without iterative refinement or tool augmentation reflects baseline model performance but may not capture the full potential of LLMs in clinical decision support. LLM outputs are inherently stochastic and may vary across sessions. Although efforts were made to standardize query conditions, response variability cannot be entirely controlled. Future studies incorporating repeated assessments over time are recommended to evaluate performance stability and potential model drift. The fourth limitation was specifying the exact model version/build is essential for ensuring replicability and enabling fair comparisons across studies, this study evaluated specific versions of LLMs available at a single point in time and did not include newer iterations (e.g., ChatGPT-4o, Gemini 2.0). Since LLMs are rapidly evolving, future research should incorporate updated models to maintain relevance and ensure comparability. Future studies should aim to document model versions or Application Programming Interface (API)-based identifiers wherever possible. The fifth limiatation was the study focused on text-based queries to reflect common real-world usage, this approach represents only one dimension of LLM capabilities. Lastly despite adherence to CHART reporting standards, certain factors such as limited transparency in model training data, evolving model updates, and inherent response variability may affect reproducibility. Certain model parameters (e.g., temperature settings and training data specifics) were neither configurable nor fully disclosed within the web-based interfaces used, reflecting platform-level limitations rather than omissions in reporting. Future research should explore multimodal interactions, including image-based inputs, to provide a more comprehensive evaluation of LLM utility in pediatric dentistry.

Recommendation for future research: Future studies should include prospective validation using larger and more diverse question sets, incorporate clinically complex and out-of-distribution scenarios, and evaluate performance within real-world caregiver populations to better establish the applicability and impact of LLMs in pediatric dentistry. The variability in effect sizes across different model specifications highlights the need for pre-registered analytical protocols in future confirmatory studies to enhance reproducibility and minimize analytical flexibility. Until such evidence is available, LLMs may serve as supportive informational tools in pediatric dentistry; however, their outputs should be interpreted cautiously and used to complement—not replace—professional clinical judgment and decision-making.

## Conclusions

This study presents an exploratory evaluation of the performance of ChatGPT-4, Gemini, and DeepSeek in generating responses to pediatric dentistry queries. While all models produced generally high-quality and reliable outputs, significant differences in performance and readability were observed. Gemini and DeepSeek outperformed ChatGPT in GQS scores. Inter-examiner agreement analysis revealed minimal systematic bias; however, variability in individual ratings and low single-measure reliability underscore the subjective nature of accuracy assessment.These findings should be interpreted with caution, as they are based on a limited set of FAQ-style questions and may be influenced by potential overlap with training data. Furthermore, the lack of patient-centered outcomes restricts the ability to draw conclusions about real-world clinical utility. These findings should be interpreted as preliminary and primarily methodological in nature, providing groundwork for future research rather than definitive clinical guidance.

## Data Availability

The original contributions presented in the study are included in the article/Supplementary Material, further inquiries can be directed to the corresponding author.
