# Supplementary material for: Feasibility and exploratory assessment of large language models for pediatric dentistry queries: a comparative study
Source: Front Oral Health. 2026 Apr 24;7:1813936. doi: 10.3389/froh.2026.1813936 (PMC13154274; doi:10.3389/froh.2026.1813936)
Supplement: Supplementary file 1 [file Datasheet1.pdf]

Supplementary Table 1: CHART (Chatbot Assessment Reporting Tool) 2025 Checklist

Section	CHART Item	Description	Reported	Location in Manuscript	Details
**Title/Abstract**	Identification	Study identified as AI chatbot evaluation	Yes	Title, Abstract	Comparative evaluation of LLMs
	Structured summary	Objectives, methods, results clearly stated	Yes	Abstract	Includes models, metrics, outcomes
**Introduction**	Rationale	Justification for evaluating AI chatbots in healthcare	Yes	Introduction	Pediatric dentistry focus
	Objectives	Clearly stated aims	Yes	Introduction	Accuracy, quality, reliability, readability
**Chatbot Description**	Model names	Name of AI systems evaluated	Yes	Methods	ChatGPT-4, Gemini, DeepSeek
	Versioning	Version/build or access date	Yes	Methods	Access date specified
	Developers	Organization/company identified	Yes	Methods	OpenAI, Google, DeepSeek
	Access method	Platform used	Yes	Methods	Web interface
**Prompt Design**	Source of prompts	How questions were developed	Yes	Methods	Online search + expert screening
	Prompt type	One-shot / iterative	Yes	Methods	One-shot standardized prompts
	Prompt reproducibility	Exact prompts reported	Yes	Supplementary	Verbatim questions provided
	Iterative prompting	Follow-up prompts used	No	Methods	Explicitly not used
**Model Configuration**	Settings	Default vs modified settings	Yes	Methods	Default settings
	Tools enabled	Plugins/browsing	Yes	Methods	Not enabled

Section	CHART Item	Description	Reporte d	Location in Manuscript	Details
	Temperature control	Randomness settings	No	Limitations	Not user-configurable through the web interface; this reflects a platform limitation rather than an omission.
**Data Collection**	Timing	Date/time of queries	Yes	Methods	15 Oct 2025
	Sample size	Number of prompts	Yes	Methods	15 questions
	Standardizatio n	Same conditions across models	Yes	Methods	Identical prompts
**Evaluation Framework**	Metrics	Outcome measures defined	Yes	Methods	GQS, DISCERN, AOI, FRES, FKGL
	Validated tools	Use of validated instruments	Yes	Methods	GQS, DISCERN
	Evaluators	Number and expertise	Yes	Methods	3 pediatric dentists
	Blinding	Independent scoring	Yes	Methods	Blinded evaluation
**Reliability Assessment**	ICC	Inter-examiner reliability	Yes	Methods/Result s	ICC reported
	Agreement analysis	Bland–Altman used	Yes	Methods/Result s	Bias & limits reported
**Statistical Analysis**	Model specification	Appropriate statistical methods	Yes	Methods	Linear mixed-effects models
	Separate analyses	Outcomes analyzed independently	Yes	Methods	GQS, DISCERN, AOI separately
	Post-hoc testing	Multiple comparison correction	Yes	Methods	Bonferroni
	Significance level	Threshold defined	Yes	Methods	$p < 0.05$
**Results**	Model comparison	Performance differences reported	Yes	Results	LMM outputs

Section	CHART Item	Description	Reported	Location in Manuscript	Details
	Reliability findings	ICC + agreement interpretation	Yes	Results	Single vs average ICC
	Effect sizes	Reported where applicable	Yes	Results	η² / Cohen's
**Reproducibility**	Method transparency	Sufficient detail provided	Yes	Methods	Standardized workflow
	Limitations	Reproducibility constraints	Yes	Discussion	Model updates, stochasticity
**Ethics**	Approval	Ethical considerations	Yes	Methods	No patient data used
	Data privacy	Patient data involvement	Yes	Methods	Not applicable
**Limitations**	Stochasticity	Model variability	Yes	Discussion	Acknowledged
	Training data contamination	Potential overlap	Yes	Discussion	Explicitly discussed
	Question validation	Instrument limitations	Yes	Discussion	No Delphi/CVI
	Temporal limitation	Single time point	Yes	Discussion	Snapshot design
**Clinical & Regulatory Context**	Regulatory discussion	FDA/SFDA context	Yes	Discussion	Expanded
	Clinical interpretation	Avoid overclaiming	Yes	Conclusion	Exploratory framing
**Conclusion**	Interpretation	Findings appropriately framed	Yes	Conclusion	Exploratory, cautious
	Future directions	Recommendations provided	Yes	Conclusion	Prospective validation
